# Resveratrol as Chemosensitizer Agent: State of Art and Future Perspectives

**DOI:** 10.3390/ijms22042049

**Published:** 2021-02-19

**Authors:** Veronica Cocetta, Vincenzo Quagliariello, Francesco Fiorica, Massimiliano Berretta, Monica Montopoli

**Affiliations:** 1 Department of Pharmaceutical and Pharmacological Sciences, University of Padova, 35131 Padova, Italy; veronica.cocetta@unipd.it; 2 Division of Cardiology, Istituto Nazionale Tumori-IRCCS-Fondazione G. Pascale, 80131 Napoli, Italy; quagliariello.enzo@gmail.com; 3 Department of Radiation Oncology, Az. ULSS 9 Scaligera, 37045 Legnago, VR, Italy; ffiorica@yahoo.it; 4 Department of Clinical and Experimental Medicine, University of Messina, 98122 Messina, Italy; 5 Veneto Institute of Molecular Medicine, VIMM, 35129 Padova, Italy

**Keywords:** resveratrol, chemosensitization, chemotherapy resistance, cancer, drug resistance, integrative medicine

## Abstract

Resistance to chemotherapy still remains a major challenge in the clinic, impairing the quality of life and survival rate of patients. The identification of unconventional chemosensitizing agents is therefore an interesting aspect of cancer research. Resveratrol has emerged in the last decades as a fascinating molecule, able to modulate several cancer-related molecular mechanisms, suggesting a possible application as an adjuvant in cancer management. This review goes deep into the existing literature concerning the possible chemosensitizing effect of resveratrol associated with the most conventional chemotherapeutic drugs. Despite the promising effects observed in different cancer types in in vitro studies, the clinical translation still presents strong limitations due to the low bioavailability of resveratrol. Recently, efforts have been moved in the field of drug delivery to identifying possible strategies/formulations useful for a more effective administration. Despite the necessity of a huge implementation in this research area, resveratrol appears as a promising molecule able to sensitize resistant tumors to drugs, suggesting its potential use in therapy-refractory cancer patients.

## 1. Introduction

In recent decades, cancer therapy and management of the disease have seen huge advances in ameliorating patient outcomes and the quality of a patient’s life. However, despite this progress, cancer still remains one of the foremost causes of mortality worldwide. The current standard treatments for cancer include surgery, radiotherapy, hormone therapy and chemotherapy (and nowadays also immunological therapies). In the last two decades, the number of therapeutic regimens has doubled annually, and to date, the Food and Drug Administration (FDA) has approved hundreds of chemotherapeutic drugs that target nucleic acids, proteins and oncogenic signaling pathways [1,2]. Undoubtedly, mortality from cancer has decreased over the past years thanks to novel treatments and early diagnosis but complications from cancer treatments are still limiting patient survival. The main reason that underlines the failure of conventional chemotherapy is the development of drug resistance. Cancer cells can be intrinsically able to elude drug toxicity or can develop drug resistance by coping with the presence of chemotherapeutic drugs, deeply impacting cancer outcomes [3]. Even if many molecular mechanisms that underpin cancer evasion of drug toxicity have been clarified, still many uncleared aspects remain. These mechanisms are made up of a diverse set of signaling pathways, which can be activated by a wealth of stimuli to promote chemoresistance [4]. The mechanisms of intrinsic or acquired resistance are multifactorial and can comprehend altered expression of intake or efflux transporters and proteins and increase ability to repair DNA damage or tolerate stress conditions, an increase of detoxification systems, defects in the apoptotic pathways, mitochondrial alterations, alteration in tumor suppressor/oncogenes and reprogramming of the metabolic pathways [4,5,6,7]. Chemoresistance is therefore a multifactorial phenomenon that is not completely understood and represents a challenge in the oncological field that needs to be explored, understood and avoided [5,7].

## 2. Natural Compounds as a Strategy for Chemosensitization

Chemosensitization is one valuable strategy to overcome chemoresistance phenomena. Chemosensitization is based on the use of one drug to enhance the activity of another by influencing one or more mechanisms of resistance. A large number of anticancer drugs available to date derive from natural compounds extracted and isolated from plants. It is known that nature is a huge source of medicine and compounds, useful for their low toxicity, low costs and affordability and also due to their multitargeting properties that allow the modulation of different signaling pathways [2,8]. Phytochemicals derived from diet contain several components that present chemopreventive properties like curcumin, silymarin, allicin, lycopene, ellagic acid and several others. Interestingly, many studies have demonstrated that some natural compounds in association with chemotherapeutic drugs can have additive/synergic effects, improving the activity of the drugs and reducing their collateral and side effects [9,10,11,12].

The interest of this review is resveratrol (RSV), a polyphenolic compound which is found in at least 72 plant species, including human dietary items, such as grapes (especially skin), blueberries, peanut, etc. [13,14]. Resveratrol (3,4,5-trihydroxy-trans-stilbene) is a phytoalexin, produced in plants as a defense mechanism in response to pathogenic attacks (fungi or bacterial infections) or environmental stress (such as UV irradiation, metallic salts, etc.) [15]. The main enzyme involved in RSV biosynthesis is the stilbene synthase, which condenses one p-coumaroyl-CoA (4-coumaroyl-CoA) and three molecules of malonyl-CoA [16]. Resveratrol is found in either cis- or trans-configurations, with the trans- form being the prominent form in nature, and the most studied [17]. It was first isolated in 1940 as an ingredient of the roots of white hellebore (Veratrum grandiflorum O. Loes), from which it derived the name (since it is a resorcinol derivative form a Veratrum species [18]), and since then it has been identified in a large number of plant species. Relatively high quantities are found in grapes, possibly because of the response of Vitis vinifera (Vitaceae) to fungal infection. Fresh grape skin contains about 50–100 μg/g [19], and in red wine, the concentration of resveratrol is about 1.5–3 mg/L [20]. For this reason, the consumption of red wine has been associated with lower incidence of heart infarction in France, with respect to other countries (the so-called French Paradox) [21]. Since the publishing by Jung et al. of the first article on the anti-cancer properties of resveratrol, a huge interest has been given to this molecule by the cancer research field [22]. Moreover, a large variety of biological effects including anti-oxidant, anti-cancer, cardio and neuroprotection, antinflammatory, etc. have been discovered and explored [16,23]. Different studies highlight resveratrol’s low toxicity and the possibility it is a subminister in relatively high doses in humans without adverse effect, making it a valuable candidate for disease management. Despite its potential beneficial activity, a major hindrance in the clinical use of resveratrol is the limited bioavailability. In vivo, RSV is absorbed by the gastrointestinal tract and it is rapidly metabolized into its 3 and 4′-O-sulate and 3-O-glucuronide conjugates less than two hours after ingestion [24]. Moreover, the intestinal flora plays a role in the metabolism of this molecule, contributing to the ratio of metabolites among different individuals. Thus, efforts have been moved to the delivery of resveratrol using nanoparticles or using different strategies like the combination with other compounds and the use of conjugated metabolites or synthetic analogues [25].

## 3. Mechanism of Action of Resveratrol in Cancer

Resveratrol has been confirmed to have a broad range of biological activities, including antioxidant, anti-viral, anti-inflammatory, anti-aging and anti-cancer [26,27,28]. A large amount of literature describes RSV as a possessor of chemoprotective effects (such as cardio and neuroprotective activity) with the ability to decrease associated side effects of chemotherapeutic agents and the ability to enhance the efficacy of drugs used for cancer treatment [29,30,31]. As mentioned before, the first published study concerning the anticancer role of resveratrol dates to 1997, when a pioneering work by Jung and colleagues underlined the efficacy of RSV in inhibiting carcinogenesis. Jang and coworkers brought to light the ability of resveratrol to inhibit the development of preneoplastic lesions in carcinogen-treated mouse mammary glands in culture and inhibit tumorigenesis in a mouse skin cancer model [22].

It is known that carcinogenesis is a multistep and multifactorial process that involves tumor initiation, promotion and progression and that it is orchestrated by numerous molecular and cellular alterations and pathways [32,33]. Resveratrol activities are various and play a role in different stages of cancer route. It prevents tumor initiation (antioxidant and antimutagen activities), reduces tumor promotion (antinflammatory activity and COX-hydroperoxides inhibition), inhibits tumor growth (by interfering with metabolic pathways like glucose metabolism, etc. [34]) and reduces the clonogenic and metastatic potential [35]. Studies suggest that resveratrol can also potentiate its antitumor effect by interfering with signaling pathways of cellular microenvironments’ components like macrophages, fibroblasts, etc. [36]. A large amount of literature of the last three decades has brought to light the impact of RSV on a variety of signaling pathways like STAT3, Akt/mTOR, Wnt signaling, the insulin-like growth factor system, SIRT1/AMPK, etc. [37]. It has been largely demonstrated that SIRT1 is a major target of resveratrol, and several studies demonstrated that SIRT1 upregulation is required for resveratrol-mediated chemopreventive effects in colorectal cancer cells [38,39]. Furthermore, resveratrol downregulates NF-KB phosphorylation and acetylation causing impairments in factors involved in tumor invasion and metastasis, and the physical interaction between NF-KB and SIRT1 indicates that NF-KB could be implicated in resveratrol/SIRT1-dependent anticancer activity in cancer cells [38,40,41].

Interestingly, RSV has been shown to explicate its anticancer therapeutic potential also by modulating non-coding RNA (short non-coding RNAs (miRNA) and long non-coding RNAs (lncRNA)) expression, thus impacting on genes involved in the malignant phenotype [42]. In fact, numerous studies demonstrated the ability of resveratrol to regulate oncogenic miRNAs like miR-19, miR-21 and miR-30a-5p, thus affecting their target genes such as p53, PTEN, STAT3, NF-KB, COX-2, etc. In addition, long non-coding RNAs have been identified as possible targets of RSV: MEG3, ST7OT1, NEAT1 and MIR55HG in glioma cell lines are upregulated by resveratrol treatment influencing tumor progression [43]. RSV reduces the expression of AK001796 lncRNA, which is over-expressed in A549 lung cancer cells [44]. The lncRNA metastasis-associated lung adenocarcinoma transcript 1 (MALAT1) is reported to be a crucial regulator in the progression of several cancers including renal, cervical, liver, osteosarcoma, etc. RSV inhibits the invasion and metastasis of CRC cell lines through MALAT-1-mediated Wnt/β-catenin signaling [45].

## 4. Resveratrol as a Chemosensitizer Agent

**Figure 1:**
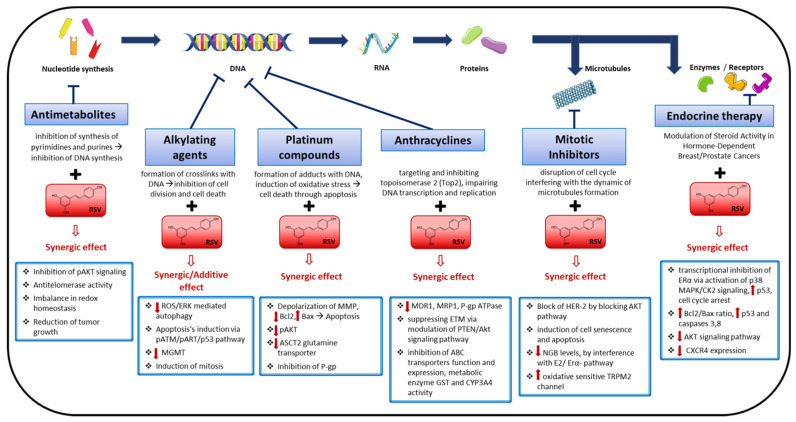
Schematic representation of the principal mechanisms involved in the chemosensitizing effect of resveratrol (RSV). The figure gives an illustrative panoramic of the principal molecular mechanisms that underpin the synergic or additive effect of resveratrol associated with the conventional antitumor therapies.

Drug resistance still remains the principal limiting factor to success in cancer patient cures. The initial solution to the problem of resistance to a single agent chemotherapy is the combined administration of agents with different mechanisms of action [46]. Moreover, collateral and side effects related to the use of a chemotherapeutic drug are other relevant factors to take into consideration. As mentioned before, chemosensitization by a molecule is a strategy that could increase drug efficacy by modulating different cell pathways. This review consists of an overview of the principal and more recent investigations focused on the chemosensitizing effect of resveratrol associated with different classes of chemotherapeutic drugs. The association can lead to an additive or synergic effect, allowing a better response to the drug treatment, potentiating the effects, lowering the doses, and reducing the collateral and side effects (Figure 1). The review aims at giving a wide panoramic of the pleiotropic effects of resveratrol in the context of chemoresistance, ranging from in vitro to in vivo. The structure of the review, divided by drug class, will allow a better comprehension of the wide range of molecular effects that underline the chemosensitizing effect of resveratrol against drugs that act with different mechanisms.

### 4.1. Alkylating Agents

Alkylating agents were among the first drugs employed to treat human cancer and still remain in use for several solid tumors. The mechanism of action is linked to their ability to act during all phases of the cell cycle on DNA, by forming crosslinks and leading to inhibition of cell division and eventually cell death. Intrinsic or acquired chemoresistance to this class of drugs is a major cause of treatment failure [47].

In the treatment of glioblastoma (GBM), the current gold standard is temozolomide (TMZ), an oral alkylating agent. The mechanism of action of TMZ consists of interaction with methyl groups on guanines and adenines in genomic DNA, with consequent induction of cell cycle arrest at G2/M phase and apoptosis. TMZ induces both apoptosis and autophagy in glioma cells through ROS and activation of the extracellular signal-regulate kinase (ERK). However, the autophagy process also exerts a protective effect, by avoiding apoptotic cell death [48]. At least 50% of patients under treatment with TMZ do not respond to the drug, mainly due to overexpression of O6-methylguanine methyltransferase (MGMT) and/or a decrease in the rate of DNA repair involving the p53 tumor suppressor MDM2 protein [49,50,51]. Moreover, ATP-binding cassette (ABC) transporters appear to be overexpressed by cells of the blood-brain barrier (BBB) and the blood-tumor barrier, concurring with the resistance phenomena [52].

It has been demonstrated that RSV increases the therapeutic efficacy of TMZ in different ways. One proposed activity is by reducing ROS/ERK-mediated autophagy, thus increasing apoptosis. In the SHG44 GBM cell line, resveratrol induces an additive antiproliferative effect to TMZ, via a ROS-dependent AMPK-TSC-mTOR signaling pathway. These results were also confirmed in in vivo xenograft mouse models, where the cotreatment induces a reduction of the tumor volume and tumor proliferation [53]. Further studies indicate that GBM-initiating cells (GICs), which display stem cell properties, are involved in tumor resistance to TMZ; RSV has been shown to enhance the sensitivity of GICs to TMZ via activation of the DNA double strands/pATM/pART/p53 pathway, inducing apoptosis [54]. Moreover, a RES dimer, ε-viniferin, has been shown to increase apoptosis of the GBM cell line, induced by cisplatin through the activation of caspase 3, 8 and 9 [55]. Another mechanism of TMZ resistance is the expression of the protein MGMT. Resveratrol reverses the TMZ resistance of GBM cells by downregulating MGMT via the NF-KB dependent pathway and via the repression of the activated Wnt signaling pathway [56,57]. Another study demonstrated that RSV pushes glioma cells treated with TMZ through mitosis, leading to mitotic catastrophe and senescence, thus improving the effect of TMZ [58].

### 4.2. Platinum Compounds

Platinum-based drugs, such as cisplatin, carboplatin, oxaliplatin, etc., are routinely used for the treatment of several solid tumors including ovarian, testicular, bladder, lung, head and neck, etc. The mechanism of action of this class of drug involves the formation of adducts between the molecules and the DNA, preventing replication and transcription and causing cell death through apoptosis, besides the induction of oxidative stress [59]. The use of these drugs is limited by the severity of the collateral and side effects and by the frequent onset of resistance due to different alterations of different molecular aspects during the course of treatment, leading to therapeutic failure and tumor relapse [60,61,62,63]. Numerous studies have focused on the effect of naturally derived compounds on platinum-resistant cancers in order to explore the potentiality of an adjuvant and integrative medicine [64]. The combination of resveratrol and platinum-based drugs presents synergic or additive effects increasing the chemosensitivity in various cancer cells, and this effect is mediated by different mechanisms of action of the phytoalexin, which cooperates with the drugs.

Resveratrol treatment could lead to depolarization of MMP, increasing the release of cytochrome c from mitochondria to cytosol, via upregulation of Bax expression and downregulation of Bcl-2, thus resulting in apoptosis. Evidence shows that combined treatment of RSV and cisplatin results in more effective inhibition of non-small lung cancer cells (NSCLC) proliferation and induction of apoptosis than the cisplatin alone [65]. In fact, this study by Ma et al. evidenced that the combination between cisplatin and resveratrol dramatically improved the efficacy of cisplatin on depolarization of MMP in H838 and H520 non-small lung cancer cells, resulting in enhanced proliferation inhibition and apoptosis [65]. Synergistic effects in combination with cisplatin have also been demonstrated in A549 lung carcinoma cells. Results show that the combination induces an enhancement in autophagy by decreasing the accumulation of autophagosome and LC3-II levels. Many reports indicate that Class I PI3Ks can activate AKT/PKB through phosphorylation, leading to inhibition of autophagy [66]. In this work by Hu et al., they evaluate the effect of the combination on AKT. Results demonstrate that resveratrol and cisplatin cotreatment decreases the phosphorylation of AKT thus inducing autophagy [67].

The impact of cotreatment with cisplatin and resveratrol has also been tested in hepatoma cells. Results in C3A and SMCC7721 cells showed that resveratrol inhibits cell growth in a dose-dependent manner and that the association with cisplatin potentiates the cisplatin-induced apoptotic effect. In hepatoma cells, this result has been linked to the lowering effect of resveratrol on glutamine absorption mediated by the reduction of the expression of glutamine transporter ASCT2. Since glutamine addiction is a major characteristic of different cancer cells and this amino acid can be directly converted into glutathione for ROS scavenging purposes [68], the study of Liu and coworkers demonstrates that resveratrol-induced chemosensitivity to cisplatin is associated with an imbalance in the redox homeostasis which fosters the DNA damage and the apoptosis. Interestingly, data indicate that resveratrol is able to inhibit the glutamine metabolism of human hepatoma cell lines increasing the toxic effect of chemotherapy, but not on normal hepatic cells [69]. Another work by Nessa et al. suggests that a prior incubation with resveratrol sensitizes A2780 ovarian cancer cells to cisplatin and oxaliplatin by downregulating NF-KB [70]. In line with this last work, another study confirmed that a 48-h pretreatment with resveratrol enhanced cisplatin cytotoxicity by a factor 3.1 in the A2780 cell line. In this study, it was also demonstrated that a permanent presence of RSV is able to prevent the development of cisplatin resistance in A2780 cell lines, but RSV treatment of the cisplatin resistant sub-clone A2780CisR, even if able to reduce cell proliferation and migration, was ineffective in reversing the cisplatin resistance [71]. The proapoptotic effect of resveratrol has also been exploited by Rezk and coworkers to sensitize OVCAR-3 cell lines to cisplatin treatment. This work gives further support to the statement that resveratrol could enhance cisplatin toxicity, underlining the importance of the timing of the treatment: resveratrol has in fact been added before cisplatin treatment so that the activation of apoptosis-related signal transduction pathways could occur before the exposure to cisplatin [72]. Further data highlight the potentiality of the combination of resveratrol and platinum-based drugs (cisplatin and carboplatin) in epithelial ovarian cancer cell lines. Results by Bjorklund et al. state that RSV co-treatment with platinum prevents the drug-free regrowth in A2780 and CaOv-4 cells, inducing irreversible growth inhibition and loss of clonogenicity. Interestingly, the association with RSV allows the use of lower doses of platinum compounds to obtain toxic effects, thus suggesting the potentiating effect of the phytoalexin [73].

Daniel and Ezekiel assert that in the colon cancer cell line, cotreatment with resveratrol and oxaliplatin showed significant inhibition of cell growth at a lower concentration than that of the single compounds alone, while this effect is not observed at higher concentrations, indicating synergism between the two molecules [74].

A work that goes against the trend states that resveratrol post treatment can nullify the toxicity of oxaliplatin in HCT116 colon cancer cells. Park demonstrates that resveratrol treatment reestablished the surviving expression levels, both mRNA and protein, in HCT116 cells, suppressed by oxaliplatin, indicating a strong recovering effect of RSV on oxaliplatin-mediated cellular toxicity [75].

Kaminski et al. investigated the effect of RSV in enhancing antitumor activity of Oxaliplatin in the Caco-2 colon cancer cell line and its possible implication in inflammatory response. Results showed that the combined treatment synergistically inhibits cell growth by interfering with caspase-3-activation, PARP cleavage and depolarization of mitochondrial membrane potential. Moreover, cotreatment prevents the immunosuppressive potential in macrophages, rendering them potentially tumoricidal [76].

An in vivo study demonstrated that treatment of EAC tumor-bearing mice with RSV enhanced the cisplatin cytotoxicity, increasing long term survival. This work also demonstrates that co-administration of RSV and cisplatin increases the intracellular levels of cisplatin. The increase in cisplatin uptake in Ehrlich cells may be explained by the effect of resveratrol in the inhibition of P-gp, which plays an important role in absorption, distribution and elimination of anticancer drugs [77]. They also evaluate the molecular bases of this result, highlighting an involvement of resveratrol in the induction of apoptosis through activation of proapoptotic family members.

Cheng et al. suggested a potentiating effect of RSV in cisplatin-induced cell death in melanoma models, through the upregulation of connexin 43 expression, which facilitates the entry of the chemotherapeutic drugs and/or transmits death signals to microenvironmental cells [78].

### 4.3. Anthracyclines

Anthracyclines, such as doxorubicin (DOX or Adriamycin), epirubicin and daunorubicin, are antibiotic molecules, highly effective in anticancer therapies. These drugs exert their anticancer action mainly by directly targeting and inhibiting topoisomerase 2 (Top2) in cancer cells, impairing DNA transcription and replication [79]. Anthracycline drugs are commonly used as treatments for malignant breast cancer resistant to endocrine therapy, gastric and bladder cancers, lymphoma, etc. Despite the efficacy of the treatment, acquired resistance is a major obstacle in clinical settings, which severely impedes the therapeutic results, leading to poor prognosis. Moreover, the occurrence of epithelial-mesenchymal transition not only increases the metastatic potential but is implicated in the onset of drug resistance [80]. As for the resistance onset, the molecular mechanisms are several and not completely elucidated. Among the others, overexpression of multidrug resistance (MDR) proteins or other plasma membrane multidrug transporters (P-gp), alteration of cellular signaling pathways and evasion of apoptosis are key factors involved in the resistance phenomena.

Doxorubicin and resveratrol cotreatment have been shown to have a synergistic effect in inhibiting the proliferation of MDA-MB-231 and MCF-7/Adr breast cancer cells. Kim et al. demonstrated that RSV treatment decreases the expression of MDR1 and MRP1 in breast cancer cell lines and decreases the drug-stimulated P-gp ATPase activity in a concentration-dependent fashion. The same group demonstrated the positive effect of the combination treatment in a MCF-7/adr cell xenograft model, where DOX+RSV significantly reduced tumor volume and expression levels of MDR1 and MRP1 [81]. Similar results were observed by Rai et al. in MCF-7 and MDA-MB-231 cell lines. The combination of doxorubicin and RSV showed potent growth inhibition and a decrease in clonogenic potential, as well as inhibition of inflammatory response, autophagic flux and induction of apoptosis. The combination was also able to decrease tumor volume in Ehrlich ascitic carcinoma cell-bearing mice, increasing the lifespan [82].

ETM-mediated drug resistance is regulated by several canonical signaling pathways, among which PI3K/Akt is extremely relevant [83]. Recent data demonstrated that acquisition of DOX resistance in SGC7901 gastric cancer cells is mediated by ETM induced by Akt aberrant activation. Resveratrol treatment synergizes with DOX in inhibiting tumor growth and preventing cell migration. In addition, RSV reverses doxorubicin resistance in gastric cancer by suppressing ETM via modulation of the PTEN/Akt signaling pathway. Analogous results were also observed in a SGC7901/DOX in vivo xenograft tumor model [84]. The effect of resveratrol on epithelial-mesenchymal transitions and the implication for doxorubicin resistance has been tested also in MCF7/Adr breast cancer cells. Results showed that the combination of DOX with RSV inhibits cell growth, promotes apoptosis and suppresses cell migration. The effect of resveratrol is linked to the modulation of the connection between SIRT1 and β-catenin; it has been shown that RSV is able to upregulate SIRT1 expression, leading to reduction of β-catenin. In this way it is able to modulate the EMT phenotype, providing a promising strategy to reduce DOX resistance phenomena [85]. Additionally, resveratrol has been shown to chemosensitize Adriamycin-resistant MCF-7 breast cancer cells to the drug, by targeting miR-122-5p, thus regulating apoptosis-inhibitory proteins Bcl-2 and CDKs [86].

An innovative study has evaluated the effect of different combinations of DOX:RSV in 3D models of pancreatic cancer, obtained using spheroids of PANC-1 pancreatic cells. Results reveal that cell viability was more affected when the DOX:RSV combination contained higher levels of RSV, and this effect is likely linked to the ability of phytoalexin to reduce the P-glycoprotein-mediated efflux of the drug [87].

The combination of DOX and RSV increases the expression of the Bax gene in HCT116 colon cancer cell lines, and RSV enhances the doxorubicin intracellular entrapment by blocking the efflux activity of the p-glycoprotein pump, thus sensitizing colorectal cancer cells to the chemotherapeutic agent [88]. El-Readi et al. also demonstrate the efficacy of combination therapy in Caco-2 colorectal multidrug-resistant cancer cells and in the CEM/ADR5000 T lymphoblastoid cell line, resistant to doxorubicin. Results showed that resveratrol influences the efflux functions via inhibition of the function and expression of ABC transporters, the metabolic enzyme GST and CYP3A4 activity and the induced apoptosis, sensitizing the cell lines to doxorubicin treatment [89].

### 4.4. Antimetabolites

The chemotherapeutic antimetabolites work via inhibition of key steps in the synthesis of pyrimidines and purines. The inhibition of these pathways results in the accumulation of building blocks for DNA, the inhibition of DNA synthesis and the eventual induction of DNA strand breaks leading to cell death. Some tumors are resistant or refractory to antimetabolites treatment, impairing the outcome of patients [90,91].

5-fluorouracil (5-FU) inhibits the activity of thymidylate synthetase during DNA replication working as an antimetabolite, inducing cell cycle arrest and apoptosis. It is used for the treatment of various cancers including gastroenteric cancers, and drug resistance is a major obstacle in the efficacy of the therapy [92,93]. Several works suggest the efficacy of resveratrol in potentiating the cytotoxic effect of 5-FU presenting chemosensitizing properties. A study conducted in colorectal cancer cells evidenced that the combined treatment increases cell cycle arrest and decreases proliferation and migration of colorectal cancer cells by inhibiting the pAkt signaling pathway [94]. Accumulating evidence highlights a non-canonical role of telomerase in modulating not only telomere elongation but also in regulating cellular reprogramming processes. The combination of RSV and 5-FU has shown anti-telomerase activity caused by the inactivation of STAT3 and blocking of STAT3 binding to the hTERT promoter site [94]. Santandreu and coworkers, similarly, evidenced synergistic interaction between resveratrol and 5-FU in colorectal cancer cell lines. They demonstrate that the synergistic effect is due to the imbalance in redox homeostasis within the cells induced by resveratrol treatment. RSV cotreatment, in fact, induced a further increase in ROS levels, linked to the inhibition of Akt and STAT3 levels [95].

RSV and 5-FU combination also inhibits B16 murine melanoma cells proliferation and migration, via regulation of levels of AMPK, COX-2, VASP and VEGF, as compared to the compounds alone. Similar results were also observed in a B16-tumors model in mice. Data show that the combination significantly reduces tumor growth compared with that in the control group and decreases the microvascular vessels in tumor angiogenesis [96]. The cotreatment is also more efficient compared to the control in reducing tumor growth in a murine model of liver [97]. Buhrmann and coworkers evidenced that resveratrol is able to reduce TNF-β-induced promotion of the survival and migration potential of colorectal cancer cells, sensitizing them to 5-FU [98]. The same group demonstrate that in colorectal cancer cells, the chemosensitizing effect of resveratrol on 5-FU treatment is linked to the inhibition of epithelial-mesenchymal transition and to the downregulation of the NF-κB pathway [99].

Gemcitabine (Gem) is used as the initial therapy in advanced pancreatic cancer (PC), but like many drugs, the development of resistance frequently happens during the initial treatment period [100]. Resveratrol has been shown to enhance the gemcitabine sensitivity of PC cells via suppression of the stemness induced by the drug and via inhibition of the lipid synthesis, obtained by downregulation of SREBP1 (key regulator in the lipid synthesis process) [101]. A study conducted in nude mice confirmed the effect of resveratrol and gemcitabine in vivo, potentiating the effect on tumor growth [102].

Another study regarding pancreatic tumors evidences the ability of RSV to sensitize human pancreatic cell lines to gemcitabine. Results suggest that RSV treatment suppresses the expression of NAF-1 (nutrient-deprivation autophagy factor-1) by inducing ROS accumulation and activating Nrf-2 signaling. Decreasing the expression of NAF-1 impairs cell proliferation and activates apoptosis in pancreatic cancer cells, enhancing the sensitivity of cells to the drug [103]. Further support for the effect of RSV in gemcitabine resistance has been given by Jiang and coworkers; they found that resveratrol acts in pancreatic cells by activating AMP-activation protein kinase (AMPK)(Thr172) and, thus, inducing YES-activated protein (YAP) cytoplasmic retention, and inhibition of its transcriptional activity. The downregulation of YAP enhanced the sensitivity of PC cells to gemcitabine [104]. In their work of 2020, Yang et al. evidenced that RSV and Gem synergically work in PC cells. Results show that Gem treatment decreased levels of VEGF-B (vascular endothelial growth factor B) and suppressed GSK3β activity within cells and that these suppressions are more marked in conditions of treatment with RSV and Gem, both in vitro and in vivo. Moreover, the association induced an increase in cell death and smaller tumor size in mice, with respect to the single treatments. These results indicate that RSV and Gem works synergically via downregulation of VEGF-B and GSK3β [105]. Gem resistance is also reduced by RSV in a human bladder cancer cell line, and results suggest that the effect is related to the modulation of ABCC2, DCK, TK1 and TK2 function and thereby increasing PARP cleavage and apoptosis [106].

### 4.5. Mitotic Inhibitors

The activity of mitotic inhibitor drugs is exerted by disruption of the cell cycle, interfering with the dynamic of microtubules formation; they promote the assembly of microtubules and prevent their depolymerization, affecting several normal cellular functions. Moreover, other mechanisms of toxicity seem to be involved in their therapeutic properties, such as enhanced phosphorylation of Bcl-2, release of tumor necrosis factor-α and an increase of TNF receptors [107]. The observed frequent inefficiency of the drug to overcome survival signals that are activated in response to the drug leads to chemotherapeutic resistance and tumor relapse. Taxanes are mitotic inhibitor molecules used in the treatment of several solid tumors, including breast, lung, ovarian and prostate cancers [37]. The efficacy of the phytoalexin resveratrol in sensitizing cancers to taxane treatments has been extensively studied.

Vinod et al. demonstrated that the introduction of resveratrol in docetaxel chemotherapy results in the synergistic induction of cell death in HER-2-overexpressing SK-BR-3 breast cancer cells [108]. HER-2 is an epidermal growth factor receptor involved in the recruitment of various proteins, which in turn leads to activation of signal transduction cascades including PI3K/AKT/mTOR and RAF-MEK-ERK pathways, providing a pro-survival environment in breast cancer cells leading to chemotherapy resistance. This study demonstrates that docetaxel-resistant cancer cells upregulated HER2 and that treatment with RSV blocks the enhancement and activation of HER-2, in addition to blocking the AKT pathway [108].

In the MDA-MB-231 breast cancer cell line, resistant to paclitaxel, RSV presents proliferation inhibitory properties, as well as the capacity of induction of cell senescence and apoptosis. The combination not only sensitizes resistant cells to the chemotherapeutic drug but also increases the susceptibility of parental cells to the drug. Interestingly, resistant clones overexpressed both P-glycoprotein (P-gp) and CYP2C8 (respectively paclitaxel efflux pump and metabolizing enzyme), providing a possible mechanism of RSV sensitization of these cells [109]. Low resveratrol doses have been shown to exert a sensitizing effect on paclitaxel-resistant non-Hodgkin’s lymphoma (NHL) and multiple myeloma (MM) cells. RSV decreases the expression of the Bcl-x antiapoptotic protein and upregulates the expression of Bax and apoptosis protease activating factor-1 (Apaf-1). Thus, these mechanisms are fundamental in the chemosensitization of cells to paclitaxel [110].

RSV has been hypothesized to interfere also with E2/Era/NGB signaling pathways. Neuroglobin (NGB) is an antiapoptotic protein upregulated by 17B-estradiol (E2) and is implicated in the ERα pathway (E2/estrogen receptor A), related to preserving cancer cell survival in stressor conditions. Cipolletti et al. demonstrate that RSV treatment is able to increase paclitaxel sensitivity of MCF-7 and T47D ERα positive breast cancer cells. This effect is linked to the ability of RSV to decrease NGB levels, via interference with the E2/ Erα- pathway [111].

A study of 2019 evidenced the synergic interaction of resveratrol on paclitaxel-induced apoptosis in DBTRG glioblastoma cell lines. This work underlines that the association of RSV improves markers of apoptosis, mitochondrial membrane depolarization, ROS levels and caspase 3 activity in DBTRG cells, with respect to paclitaxel alone. The synergic effect seems to be mediated by the stimulation and activation of the oxidative sensitive TRPM2 channel [112].

### 4.6. Endocrine Therapy

Many human hormone-dependent cancers, such as breast, prostate, ovarian, etc., are deeply influenced by steroid hormones through various mechanisms mediated by steroid receptors [113,114]. Despite the efficacy of endocrine therapy, which inhibits steroid receptor signaling, many patients with early disease develop endocrine resistance. Alterations in the transcriptional activity of estrogen receptors (ER) and androgen receptors (AR), coupled with tumor heterogenicity, are factors implied in endocrine therapy resistance [115]. Selective estrogen receptor (ER) modulators (SERMs) like Tamoxifen, Raloxifene, etc. are used in breast cancer therapy, but despite their initial response, many patients with early disease develop endocrine resistance [116]. Similarly, among the therapies for prostate cancer, the use of androgen receptor antagonists or modulators is prominent in the treatment of this pathology, but unfortunately, many cancers develop drug resistance, hampering the effectiveness of the therapy.

Resveratrol has been considered a phytoestrogen thanks to its potent estrogenic activity initially demonstrated in MCF-7 mammary cancer cells [117]. Nevertheless, further studies with this cell line demonstrated antagonist activity of RSV in the presence of E2 [118]. The biphasic activity of phytoestrogen has been underlined during cancer development in estrogen-sensitive tissues: at the earlier stages, phytoestrogen is able to downregulate cell growth by activating ER-β, and in later stages, it can promote proliferation of cells that exhibit a high amount of ER-α but little ER-β. RSV has shown antagonist activity only for ER-α and not for ER-β subtypes. De Amicis et al. demonstrate that RSV inhibits human breast cancer cell proliferation, including MCF-7 Tamoxifen-resistant cancer cells. The mechanism concerns blockade of the cell cycle, activation of p38 MAPK/CK2 signaling and induction of p53 expression, which lastly leads to transcriptional inhibition of ERα [119]. RSV reduced ER expression in MCF-7-TR breast cancer cells resistant to the ER antagonist 4-hydroxytamoxifen (4-OHT) through several mechanisms. Specifically, they underlined the effect of RSV in activation of p38 MAPK/casein kinase II signaling and induction on p53, which recruits at the ERα proximal promoter, leading to inhibition of ER transcription. [119]. Multiple other works, marked the ability of RVS, in combination therapy, to restore ER expression via epigenetic mechanisms [120].

The synergic effect of RSV and SERMs has been demonstrated also with Raloxifene. The association increased the Bcl2/Bax ratio and expression of p53 and caspases 3,8, thus increasing apoptosis, indicating a stronger therapeutic effect with respect to the molecules alone in breast cancer cells. Results were also more evident in estrogen receptor positive MCF7 cells compared to MDA-MB-231 cells [121].

In an analogous manner like the one observed in ERα-positive breast tumor cells, RSV inhibits DNA synthesis and modulates cell cycle progression in androgen receptor positive prostate cancer cells [122]. Androgen deprivation therapy using Bicalutamide or Enzalutamide, which are androgen receptor antagonists, are commonly used as a therapeutic drug for prostate cancers. Studies suggest a chemosensitizing role of resveratrol in prostate cancer cells treated with chemotherapy drugs. Jang et al. in 2019 evidenced that RSV and its combination with Bicalutamide or an antagonist of CXCR4, effectively suppressed prostate cancer progression via downregulation of the AKT signaling pathway [123]. CXCR4 is a chemokine receptor commonly upregulated in several cancers including prostate cancers and, together with androgen receptors, has been implicated in the promotion of PCs progression [124]. Resveratrol has also been previously shown to suppress CXCR4 expression [125]. Another study performed on LNCaP and hormone resistance LNCaP-B underlined the effect of RSV. In this study, it was demonstrated that RSV induces apoptosis in LNCaP-B cells via decreasing the expression of ARV7 (androgen receptor splice variant-7), which is overexpressed in the resistant cells, and inhibiting the activation of the AKT pathway [126].

## 5. Conclusions and Perspectives

In preclinical trials resveratrol has shown immense potential in the cancer field, demonstrated to be an ideal candidate as a chemopreventive/adjuvant/chemosensitizing agent. A positive property of this molecule is that it is well-tolerated in patients and appears to induce minimal collateral and side effects even at high doses; this aspect has been validated by several clinical trials aimed at studying the safety, pharmacokinetics and metabolism of resveratrol [27]. However, some participants reported gastrointestinal symptoms. A large number of clinical trials have been performed or are ongoing to evaluate the effect of this molecule in neurological disease and cognitive performance, diabetes, cardiovascular disease, obesity, cancer and other conditions related to oxidative stress and inflammation. However, to the best of our knowledge, the studies in the cancer field are focused on the chemopreventive effect of the molecule, and in certain types of cancer, resveratrol had unclear and sometimes even detrimental effects [127]. It is of fundamental relevance to underline that the contrasting effect observed in resveratrol studies can be explained by several factors like the number and characteristics of the patients, the health status of the gut microbiota, the dose, the type of administration and the medium (food or without food). All these factors may influence the effect of the administration, and for this reason, a deeper investigation has to be performed.

One of the main problems in the clinical translation of this molecule is the limited bioavailability since resveratrol is fast metabolized and eliminated by the body, rendering difficult the maintenance of a therapeutically relevant concentration in the bloodstream [128]. Several approaches are currently in use to overcome this problem, integrating expertise in biology, chemistry, pharmaceutical technology, etc. In addition to the use of naturally occurring or synthetic analogues of resveratrol, the use of conjugated metabolites, and the combination with natural agents able to inhibit in vivo resveratrol’s metabolism, in the formulation of a novel drug delivery system is of increasing interest [25]. The anticancer effect of drug delivery nanoformulation of RSV alone or in combination with other molecules has been demonstrated by several in vitro and in vivo studies in different cancer types [129].

Zhao et al. propose a co-encapsulation of DOX and RSV in poly (lactic-co-glycolic acid) (PLGA)-based nanoparticles (NPS). This strategy prolonged the half-life of both DOX and RSV, increased the concentrations of both the molecules within the tumor tissue while reducing the toxicity of DOX in healthy tissue, and presented efficacy in overcoming DOX resistance [130]. Their studies have been performed in breast cancer cells and in tumor-bearing mice. Data indicate that DOX/RES-loaded nanoparticles were simultaneously delivered in the nucleus of MDA-MB-231/ADR cells and MCF-7/ADR resistant breast cancer cells, allowing them to overcome chemotherapy resistance by inhibiting P-gp, MRP-1 and BCRP resistance proteins, and by inducing apoptosis by modulating NF-KB and Bcl-2. In in vivo experiments, the DOX/RSV-loaded NPS mainly delivered the drugs to tumor tissue, inhibiting DOX-resistant tumor growth and presenting lower levels of systemic toxicity [130]. Another technology for encapsulation is represented by liposomes, which have gained FDA approval in clinical use (like liposomal formulations of doxorubicin and daunorubicin for metastatic breast cancer and AIDS-related Kaposi’s sarcoma). A study by Meng et al. evaluated the effect of a PEGylated liposome charged with resveratrol and paclitaxel in multidrug-resistant tumor cells and in vivo models. The in vitro study, in resistant MCF-7/Adr tumor cells, showed that the liposomal formulation exhibits potent cytotoxic activity, overcoming multidrug resistance against the resistant cells. In the in vivo section of this work, they demonstrated that combination could enhance bioavailability and tumor retention of the drugs; moreover, the composite treatment in liposomal formulation significantly inhibits drug-resistant tumor in mice, without notable effects in the systemic toxicity. Thus, these results suggest that the composite liposomes improve the treatment of both drug-resistant and drug-sensitive tumors, generating a synergistic anticancer effect on the tumor [131].

Lowering the doses of chemotherapeutic drugs and combining them with chemopreventive or chemosensitizing agents may be a valuable strategy not only to decrease the toxicity of the drugs but also to increase the efficiency of traditional chemotherapeutic regimens. This approach could be particularly relevant in tumors difficult to treat due to pharmacological resistance, which is fostered by different molecular mechanisms. Complementary and alternative medicine (CAM) is largely believed to present an integrative role within conventional therapy even in the context of cancer therapy, sustaining natural products employment [132,133,134,135,136]. Resveratrol represents a highly promising dietary phytochemical, with interesting properties in the context of different pathologies, including cancers. Among a large amount of literature aimed at the study of its pleiotropic effect, identification of the molecular mechanism of action and the possible use in treatment, cancer chemosensitization has gained large attention. As highlighted in this review a large number of studies have explored the potentiality of resveratrol to sensitize cancer cells to drugs belonging to different chemotherapeutic classes (summarized in Table 1). This molecule appears to enhance anticancer therapies by regulating multidrug-resistant protein expressions, by interfering with cell signaling pathways and cell cycle regulators and by influencing apoptosis [8]. Despite the promising efficacy observed in a large number of in vitro and in vivo studies, the clinical translation in oncology still presents ambiguous results. Thus, more clinical data are necessary to understand the potential of resveratrol’s therapeutic use. Moreover, the low bioavailability represents a big challenge in the pharmacological use of this molecule and further studies are necessary to optimize nanocarrier delivery systems of strategies to improve it. Specifically, nanomedicine will be the key factor in solving the problem of the low oral bioavailability of resveratrol. Several strategies are currently available in commerce based on lipid nanocarriers, for example, curcumin/piperine-loaded liposomes (i.e., Meriva products) [137]; in fact, as demonstrated in clinical trials, these formulations significantly increased the anti-inflammatory properties of curcuminoids compared to the unformulated ones through a proper resistance to the acidic pH of the stomach associated to a better absorbance in the small intestine probably exploiting biliary emulsions, as happens for many lipophilic molecules [138]. Other innovative nanoemulsions are currently under study [139] and will most likely be the subject of interesting implications in the clinical field also to optimize the resistance and biodistribution of resveratrol.

## Figures and Tables

**Table 1:** Summary of the current literature concerning the chemosensitizing effect of resveratrol combined with conventional anticancer drugs.

Drug Class	Drug	Cancer Model	Effect in Combination with Resveratrol	References
Alkylating agents	Temozolomide	SHG44 GBM cell line In vivo xenograft mouse models	Additive effect by ROS-dependent AMPK-TSC-mTOR signaling pathway Reduction of tumor growth	[53]
		GBM-initiating cells (GICs)	Activation of the DNA double strands pATM/pART/p53 pathway	[54]
		GBM cells	By downregulating MGMT via the NF-KB dependent pathway and via the repression of the activated Wnt signaling pathway	[56,57]
		Glioma cells	Mitotic catastrophe and senescence	[58]
Platinum compounds	Cisplatin	Non-small lung cancer cells (NSCLC) H838 and H520	Depolarization of MMP and apoptosis	[65]
		A549 lung carcinoma cells	Decreases the phosphorylation of AKT thus inducing autophagy	[67]
		Hepatoma cells C3A and SMCC7721	Reduction of transporter ASCT2 imbalance in the redox homeostasis	[69]
		A2780, OVCAR-3 CaOv-4	Proapoptotic effect	[70,71,72,73]
		EAC tumor-bearing mice	Activation of proapoptotic family members	[77]
		Melanoma models	Upregulation of connexin 43 expression	[78]
	Oxaliplatin	Colon cancer cell line	Inhibition of cell growth	[74]
		Colon cancer cell line Caco2	Interfering with caspase-3-activation, PARP cleavage, and depolarization of mitochondrial membrane potential	[76]
		HCT116 cells	Reestablishment of surviving protein expression	[75]
Anthracyclines	Doxorubicin (Adriamycin)	MDA-MB-231 and MCF-7/Adr breast cancer cells MCF-7/Adr cell xenograft model	Decreases the expression of MDR1 and MRP1; decreases P-gp ATPase activity Reduces tumor volume and expression levels of MDR1 and MRP1	[81]
		MCF-7 and MDA-MB-231 cell lines Ehrlich ascitic carcinoma cells-bearing mice	Growth inhibition, decreased clonogenic potential, inhibition of inflammatory response, induction of apoptosis. Reduced tumor volume, increased lifespan	[82]
		SGC7901 gastric cancer and SGC7901/DOX in vivo xenograft tumor model	Suppressing ETM via modulation of PTEN/Akt signaling pathway	[84]
		MCF7/Adr breast cancer cells	Regulation of SIRT1/ β-catenin pathway	[85]
		MCF-7/Adr breast cancer cells	Targeting miR-122-5p, thus regulating apoptosis-inhibitory proteins Bcl-2 and CDKs	[86]
		Spheroids of PANC-1 pancreatic cells	Reduced P-glycoprotein mediated efflux of the drug	[87]
		HCT116 colon cancer cell lines	Increased expression of Bax gene and blocked the efflux activity of p-gp	[88]
		Caco-2 colorectal and CEM/ADR5000 T lymphoblastoid cell line	Inhibition of the ABC transporters, metabolic enzyme GST and CYP3A4 activity	[89]
Antimetabolites	5-Fluorouracil	Colorectal cancer cells	Inhibiting pAkt signaling pathway Reduction of TNF-β-induced epithelial-mesenchymal transition and downregulation of NF-KB	[94] [98,99]
		Colorectal cancer cell lines	Imbalance in redox homeostasis linked to inhibition of Akt and STAT3 levels	[95]
		B16 murine melanoma cells B16-tumors model in mice	Regulation of levels of AMPK, COX-2, VASP and VEGF Reduces tumor growth	[96]
		Murine model of liver	Reduces tumor growth	[97]
	Gemcitabine	Advanced pancreatic cancer (PC)	Downregulation of SREBP1	[101]
		PaCa xenografts in nude mice	Reduces tumor growth	[102]
		Pancreatic cancer cells	ROS accumulation, activation of Nrf-2 signaling, suppression of NAF-1	[103]
		Pancreatic cancer cells	Downregulation of YAP	[104]
		Pancreatic cancer cells and in vivo model	Downregulation of VEGF-B and GSK3β	[105]
		Human bladder cancer cell line	Modulation of ABCC2, DCK, TK1 and TK2 function and increased PARP cleavage and apoptosis	[106]
Mitotic Inhibitors	Docetaxel	HER-2-overexpressing SK-BR-3 breast cancer cells	Blocks the enhancement and activation of HER-2, in addition to blocking AKT pathway	[108]
	Paclitaxel	MDA-MB-231 breast cancer cell line	Induction of cell senescence and apoptosis	[109]
		Paclitaxel-resistant non-Hodgkin’s lymphoma and multiple myeloma	Decreases Bcl-x, upregulates Bax and Apaf-1 expression.	[110]
		MCF-7 and T47D ERα positive breast cancer cells	Decreases NGB levels, via interference with E2/Erα pathway	[111]
		DBTRG glioblastoma cell lines	Stimulation and activation of the oxidative sensitive TRPM2 channel	[112]
Endocrine therapy	Tamoxifen	MCF-7 Tamoxifen-resistant cancer cells	Transcriptional inhibition of ERα via blockade of cell cycle, activation of p38 MAPK/CK2 signaling and induction of p53	[119]
	4-hydroxy -tamoxifen (4-OHT)	MCF-7-TR breast cancer cells	Inhibition of ER transcription via p38 MAPK/casein kinase II signaling, p53, binding with the transcription nuclear factor Y (NF-Y) to the ER proximal promoter	[119]
	Raloxifene	MCF7 cells estrogen-receptor positive	Increases the Bcl2/Bax ratio and expression of p53 and caspases 3,8	[121]
	Bicalutamide	Prostate cancer	Via downregulation of AKT signaling pathway, suppresses CXCR4 expression	[123,125]
